# Neonatal nasogastric tube feeding in a low-resource African setting – using ergonomics methods to explore quality and safety issues in task sharing

**DOI:** 10.1186/s12912-018-0314-y

**Published:** 2018-11-16

**Authors:** Gregory B. Omondi, George Serem, Nancy Abuya, David Gathara, Neville A. Stanton, Dorothy Agedo, Mike English, Georgina A. V. Murphy

**Affiliations:** 1 KEMRI-Wellcome Trust Research Programme, Nairobi, Kenya; 2 Nairobi City County Government, Nairobi, Kenya; 3 Faculty of Engineering and the Environment, University of Southampton, Southampton, UK; 4 Kenyatta National Hospital, Nairobi, Kenya; 5 Nuffield Department of Medicine, University of Oxford, Oxford, UK

**Keywords:** Ergonomics, Task analysis, Quality and safety, Nasogastric tube feeding

## Abstract

**Background:**

Sharing tasks with lower cadre workers may help ease the burden of work on the constrained nursing workforce in low- and middle-income countries but the quality and safety issues associated with shifting tasks are rarely critically evaluated. This research explored this gap using a Human Factors and Ergonomics (HFE) method as a novel approach to address this gap and inform task sharing policies in neonatal care settings in Kenya.

**Methods:**

We used Hierarchical Task Analysis (HTA) and the Systematic Human Error Reduction and Prediction Approach (SHERPA) to analyse and identify the nature and significance of potential errors of nasogastric tube (NGT) feeding in a neonatal setting and to gain a preliminary understanding of informal task sharing.

**Results:**

A total of 47 end tasks were identified from the HTA. Sharing, supervision and risk levels of these tasks reported by subject matter experts (SMEs) varied broadly. More than half of the tasks (58.3%) were shared with mothers, of these, 31.7% (13/41) and 68.3% were assigned a medium and low level of risk by the majority (≥4) of SMEs respectively. Few tasks were reported as ‘often missed’ by the majority of SMEs. SHERPA analysis suggested omission was the commonest type of error, however, due to the low risk nature, omission would potentially result in minor consequences. Training and provision of checklists for NGT feeding were the key approaches for remedying most errors. By extension these strategies could support safer task shifting.

**Conclusion:**

Inclusion of mothers and casual workers in care provided to sick infants is reported by SMEs in the Kenyan neonatal settings. Ergonomics methods proved useful in working with Kenyan SMEs to identify possible errors and the training and supervision needs for safer task-sharing.

**Electronic supplementary material:**

The online version of this article (10.1186/s12912-018-0314-y) contains supplementary material, which is available to authorized users.

## Background

Neonatal mortality has fallen more slowly than child mortality in the past twenty years in many low- and middle-income countries (LMICs) due to challenges with the provision of high quality care given the resource limited nature of such settings [1]. To improve neonatal survival, the provision of high quality care to small and sick is must improve [2]. Assisted feeding, often by nasogastric tube (NGT), is one of a set of interventions that form an essential package of facility based services. When fully implemented, feeding (oral or nasogastric) has the potential to substantially reduce neonatal mortality and morbidity, especially for low-birth-weight neonates [3]. NGT feeding is typically the formal responsibility of nurses. It is a time-consuming task that may need to be performed every two to three hours for small and sick babies [4]. In resource-limited settings, where the nursing workforce is severely constrained, components of the NGT feeding task may be only partly performed or completely missed, negatively impacting survival and early post-natal growth [5–7].

Task shifting/sharing has been proposed as an approach for addressing health workforce shortages [8–11]. However, despite the recent launch of task-sharing policies in Kenya, there are no specific guidelines that encompass task sharing between nurses and non-professional cadres in newborn units and no recognised ‘healthcare assistants’ within Kenyan public health facilities [12]. Anecdotal information suggests however, that nurses informally share tasks with untrained casual workers and babies’ family members. The safety and quality of care provided under such conditions is a major concern [13–15]. How key neonatal nursing interventions are performed and shared, which components may be missed, and what safety issues need to be considered when performing and sharing tasks, remain undescribed in such settings.

Given the importance of NGT feeding, its time-consuming nature and the potential risk of serious consequences (for example aspiration) if incorrectly performed, it is imperative to consider safety in cases where it is shared. Our aim was, therefore, to explore this task in detail, gain preliminary information on how it is shared in Kenyan public hospitals and examine potential risks. This will provide preliminary data to conduct a larger study with a larger sample. Knowledge gained will inform discussions on whether and how this task could be formally and safely shared. We employed Ergonomics (or human factors and ergonomics, HFE) methods often helpful in unpacking complexities in the dynamics of task implementation processes.

The Human Factors and Ergonomics Society defines Ergonomics as “…the scientific discipline concerned with the understanding of interactions among humans and other elements of a system, and the profession that applies theory, principles, data and methods to design in order to optimize human well-being and overall system performance.” [16] HFE methods have been traditionally used to improve quality and eliminate errors in various industries predominantly the aviation, nuclear, manufacturing and oil and gas industries [17]. In healthcare, HFE has the potential to make work practices simpler and therefore have a direct impact on the quality of care provided [18]. A number of studies have looked at how HFE methods can be used to gain insights into the dynamic nature of patient care, improve patient safety, analyse problems to generate solutions, calculate/predict risk levels as well as design solutions to mitigate medication administration errors. However, others argue that HFE methods are currently underutilised in healthcare in exploring issues of quality and safety [15, 19, 20].

In this study, we use Hierarchical Task Analysis (HTA) which is a flexible and structured technique to provide an exhaustive description of tasks in a hierarchical manner [21], and the Systematic Human Error Reduction and Prediction Approach (SHERPA) to describe the errors that might occur in each step of the HTA, the consequences, probability and criticality of such errors, and the remedial steps to be taken to reduce them [21, 22]. Healthcare Failure Mode and Effects Analysis (HFMEA) is a similar method to HTA and SHERPA and has also been used to identify potential failures and their causes before future services are provided and/or to improve current services. While both methods have the ultimate goal of improving patient safety, HFMEA has been shown to have validity challenges [23, 24]. SHERPA’s reliability and validity is consistently high, ranging between 0.65–0.9 and 0.74–0.8, respectively, and higher than other human error identification techniques [25–27].

## Methods

### Subject matter experts

Data collection for this study was conducted and facilitated by three researchers with direct experience in providing care, including NGT feeding, to sick and or premature infants in inpatient neonatal care settings in Kenya. Two of these researchers (GBO, GS) are registered nurses and one (NA) is a medical doctor; all were trained on HTA and SHERPA techniques by a Professor of ergonomics (NS).

Two groups of subject matter experts (SMEs) composed of four nurses (SME1) and eight nurses (SME2), respectively, were constituted for this study. Experts were purposefully selected based on their experience in frontline neonatal nursing practice, teaching and advisory roles on neonatal nursing care policy. SME members across both groups were drawn from five public sector facilities in Nairobi and Kiambu counties in Kenya admitting between 300 and 4500 babies to their neonatal units each year. Such public facilities are characterised by high patient to nurse ratios and provide the great majority of inpatient neonatal care to Kenya’s population, especially its poor. (*Murphy* et al*, PLoS One, under review*).

### Hierarchical task analysis (HTA)

**Fig. 1:**
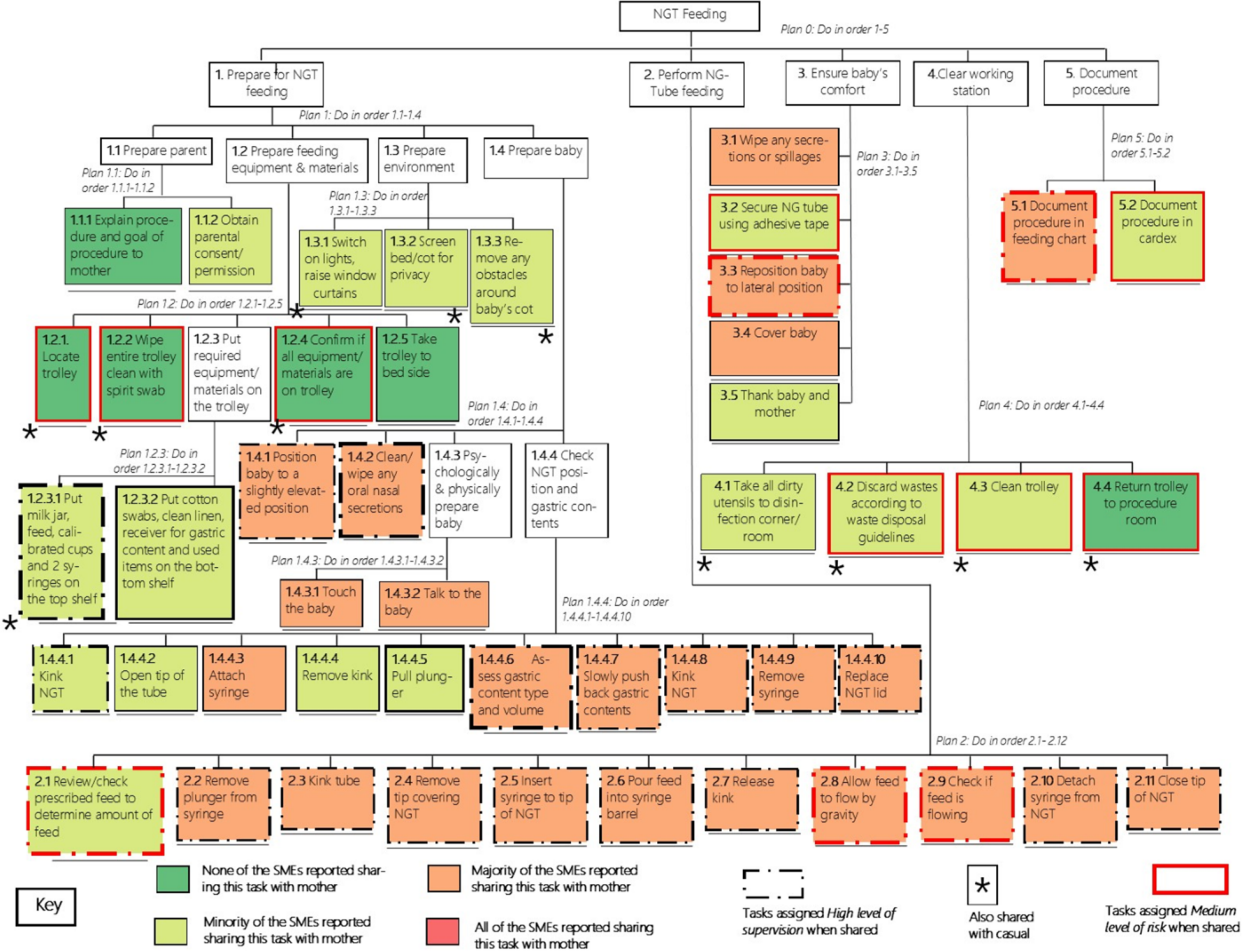
Hierarchical task analysis (HTA) of nasogastric tube (NGT) feeding in neonatal care settings in Kenya with colour codes showing sharing levels and patterns showing supervision and risk distribution

HTA was initially used by three trained researchers to create a detailed description of the tasks and sub-tasks performed by nurses while carrying out NGT feeding for sick infants. This was done based on their professional experience and information from the Manual of Clinical Procedures, 3rd Edition by the Nursing Council of Kenya [28]. Standard guidelines on performing HTA were followed [19, 22]. Briefly, the purpose of the analysis was defined and boundaries were set; system goals and sub-goals were described for the NGT feeding task; and the goal was then broken down into sub-goals with emerging operations/actions identified at each step [29]. The researchers aimed to limit the number of sub-goals under any super-ordinate goal to between 3 and 10 by grouping them into clusters of operations. The description of sub-goals ceased after consensus among the researchers was reached that the operations has become sufficiently detailed for the intended purpose of describing NGT feeding. The final operations of a sub-goal that were not broken down any further are referred to as ‘end tasks’. Further analysis was based on these end tasks (tasks in boxes with a line under them) and not the super-ordinate tasks (tasks in unshaded boxes, *see* Fig. 1).

SME1 was taken through a day-long workshop on ergonomics methods facilitated by the researchers. They were introduced to the concept of ergonomics in health and taught how to conduct a HTA and validate HTA outputs. SME1 was presented with the draft NGT feeding HTA to review and propose changes. The review was done in pairs, suggested changes were then discussed among all SMEs until consensus was reached on a relevant, clear and meaningful final version [30].

### Systematic human error reduction and prediction approach (SHERPA)

SHERPA analysis was done on the HTA end tasks by the three researchers [30]. One researcher (GBO) led the drafting of the first version, this was then reviewed iteratively by the other two researchers (GS and NA) until a consensus was reached for the final draft. For each of the tasks, the error mode/code, description, consequence, recovery, probability, criticality and remedial measures were formulated (*see* Additional file 1*:* Table S2). For the error modes/codes, SHERPA’s predefined human error taxonomy and associated codes classified under the six behaviour categories were used [19]. Probability and criticality levels used were defined as shown in Table 1 [31]. This version of SHERPA was reviewed by an expert neonatal nurse trainer (DA), changes made, and thereafter, adopted as the final version of SHERPA for the NGT feeding HTA.

**Table 1:** Definition of probability, criticality and supervision levels

Task attribute	Low	Medium	High
Probability	Never known to happen	Known to happen occasionally	Known to happen frequently
Criticality/risk	No risk of injury to patient if task is incorrectly done or missed	Risk of minor injury to patient if task is incorrectly done or missed	Risk of serious injury or death of patient if task is incorrectly done or missed
Supervision^a^	Supervision done by the nurse for only part of the implementation process of the task	Supervision done by the nurse for about half of the implementation process of the task	Supervision done by the nurse for the entire implementation process of the task

^a^During analysis, ‘low’ and ‘medium’ levels of supervision were combined into one category of ‘low/medium’

### Classification of supervision, sharing and risk level of tasks

SME2 was convened for a day-long workshop with the aim to discuss and share their expert opinion on the usual practice of NGT feeding within their settings. This did not aim to reach consensus but instead to illustrate possible variations in opinion and practice, recognising that there were only two representatives from each of the four public hospital settings and that any findings may not be regarded as representative of the wider health system in Kenya. SME2 members were asked to: i) give their opinions on who they share NGT feeding tasks with. They were then asked assign predefined levels of supervision (low, medium or high) to each of the 47 end tasks in the event they shared the task. They were also asked to consider the risk level that would occur if a task was incorrectly implemented and to then assign those levels to each of the 47 NGT feeding end tasks (see Table 1) and to state how often they considered tasks were missed (never, rarely or always) during routine care within their hospitals (Additional file 2).

For sharing purposes, mothers were defined as the guardian looking after the infant during its inpatient care. Students were defined as those taking practicum/attachment sessions in the facility while still studying towards their nursing diplomas/degrees, and casuals as non-professional personnel without any nursing or health background contracted or employed on a temporary basis to provide auxiliary services, such as cleaning. Tasks were considered missed if they were not done by the nurse, student, casual or mother. SME2 used the same definitions for risk levels as those used by the researchers when describing criticality during SHERPA (see Table 1). Our results focus on the risk levels assigned by SME2.

### Data analysis

#### HTA

The final model of the HTA was illustrated using Microsoft Publisher. A database of the sharing, missed tasks, supervision and risk level responses as reported by SME2, was created in MS Excel and imported into R Version 3 for analysis. Only descriptive statistics were used in analysing data for this study.

#### Sharing, supervision and risk levels

Simple descriptive statistics were used to calculate the proportion of tasks shared with mothers, students and casuals, for example 6 out of the 47 tasks shared would equate to 12.8% of the tasks. Responses from SME2 on sharing, supervision and risk levels were thereafter organised into four response groups according to the number of SME2 members reporting these levels. If, for example, a task was reported to be shared with the mothers by none of the SMEs, then for the purpose of analysis, that task was considered as shared by ‘none’. However, if the task was reported as shared with the mother by between one and three; four and seven, and all eight SMEs, then it was considered as shared by a ‘minority’,’ majority’ and ‘all’ SME2 members, respectively. These considerations were applied to supervision and risk levels alike and are used to report findings for this study. Colour codes are used to show how SME2 reported sharing the 47 end tasks with the mother. During analysis for shared tasks, whether a task was ever shared was of interest, hence the analysis also focuses on those tasks reported as shared by at least one SME2 (‘ever shared’).

#### Missed tasks

Tasks reported by the SME2 as missed were those tasks considered as not done at all (by either the nurse, mother or casual) during the implementation of NGT feeding. The frequency with which the tasks were missed was measured on a three-point Likert scale of ‘never missed’, ‘rarely missed’ and ‘often missed’. For analysis, tasks reported as ‘never missed’ and ‘rarely missed’ were grouped in a single ‘never/rarely missed’ category and our analysis was focussed on tasks that were reported as often missed by at least one of the SME2 members.

### Ethical consideration

Ethical approval for this study has been granted by the Kenya Medical Research Institute (KEMRI) Scientific and Ethics Review Unit (protocol No.3366).

## Results

### Hierarchical task analysis

Figure 1 shows the final NGT feeding HTA comprising one goal and five sub-goals and a total of 47 end tasks (i.e. those at the bottom of the hierarchy/tasks that are not described in further sub-tasks). Subsequent results and analysis hereafter focus on these 47 end tasks.

### Sharing

Sharing tasks with mothers and students was commonly reported by SME2 members as compared to sharing with casuals (57.3, 39.6 and 7.3% of tasks on average, respectively). All tasks were reported by at least one member of SME2 as ever shared with the students. Nearly all tasks (41/47) were reported as ever shared with the mothers whereas only a small proportion of tasks (23%, *marked with an asterisk in* Fig. 1) was reported as ever shared with the casuals. For six end tasks, none of the SME2 members reported sharing those with the mother.

There was considerable heterogeneity in the tasks reported as shared with mothers, with no task being reported as shared by all eight members of SME2 (Table 2). Slightly more than half (51.1%, *n* = 47) of the tasks were reported as shared with the mothers by a majority of SME2 members.

**Table 2:** Proportions of task sharing with mothers, students and casuals by subject matter expert group 2

		Proportions of tasks shared with:		
Subject matter experts	Current care setting	Mother	Student	Casual
Expert 1a	County referral hospital, approx. 300 annual neonatal admissions, 2 nurses on a typical day shift,15 cots and 52% average occupancy	12.8%	NA^a^	14.9%
Expert 1b		51.1%	NA^a^	8.5%
Expert 2a	Large maternity hospital, approx. 4200 annual neonatal admissions, 3 nurses on a typical day shift, 63 cots and 73% average occupancy	55.3%	63.8%	14.9%
Expert 2b		19.1%	97.9%	0.0%
Expert 3a	County referral hospital, approx. 1800 annual neonatal admission, 2 nurses on a typical shift, 40 cots and slightly above 100% average occupancy	53.2%	61.7%	12.8%
Expert 3b		12.8%	95.7%	6.4%
Expert 4a	National referral hospital, approx. 3200 annual neonatal admissions, 9 nurses on a typical day shift, 56 cots and 117% average occupancy.	61.7%	0.0%	0.0%
Expert 4b		57.4%	31.9%	2.1%

^a^Not applicable (NA): No students come for practicums or are taught at this facility

### Missed tasks

Four tasks were reported as often missed by the majority of SME2 while the remaining tasks (91.5%, *n* = 47) were reportedly never/rarely missed. Those tasks reported as often missed were under the sub-goals ‘prepare for NGT feeding’, and ‘ensure infant’s comfort’ (see Additional file 3: Figure S1*)*. There was clear consensus among all SME2 that 16 of the 47 tasks were never/rarely missed, while for the remaining 31 tasks at least one SME2 member reported the task was often missed.

### Risk levels

Overall, there was considerable heterogeneity in the reported levels of risk by SME2. Thirteen tasks were assigned medium level of risk by the majority (≥ 4 of 8) SME2 members. These 13, plus an additional five tasks, were assigned medium level of criticality by the researchers during SHERPA analysis (see Additional file 4: Table S1), demonstrating high concordance in the levels of risk assigned to the tasks by the two groups. None of the 47 tasks were given a high criticality rating by the researchers or SME2 members.

### Overlap between task sharing, risk levels and missed tasks

**Fig. 2:**
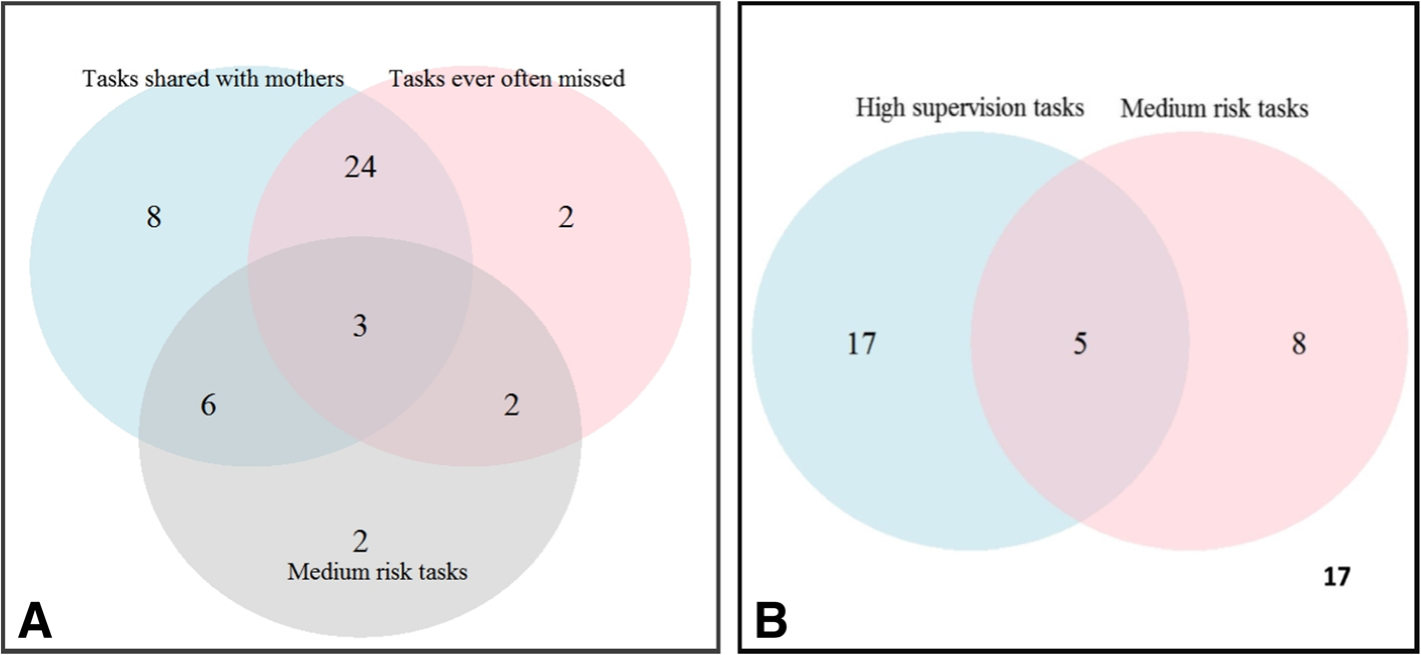
**a** Venn diagram showing the overlap between tasks reported as shared and often missed by at least one of the SME, and tasks assigned medium risk level by the majority of SMEs. **b** Venn diagram showing the overlap of tasks reported as highly supervised and those assigned medium risk level by the majority of SMEs. 17 tasks were neither assigned a high supervision nor medium risk level

Nine tasks (22%) reported as shared with the mother by at least one of the SME2 were assigned a medium level of risk by a majority (≥ 4 of 8) of SME2 members. Of the nine tasks, three were under ‘perform NGT feeding’, two under ‘ensure baby’s comfort’, two under ‘clear working station’ and two under ‘document procedure’. Twenty-seven (27/41) of the tasks reported as shared with the mother by at least one SME were also reported to be missed by at least one SME (see Fig. 2a).

### Supervision

Considerable heterogeneity was observed in the reported supervision and risk levels of the tasks reported as shared with mothers. Twenty-two tasks were assigned high supervision levels by the majority (≥4 of 8) of SMEs; of these tasks, five were reported as having medium risk (see Fig. 2b). Those tasks reported as highly supervised were predominantly under the sub-goals ‘perform NGT feeding’ (11/22) and ‘prepare for NGT feeding’ (9/22).

### SHERPA

The most common type of error mode assigned was ‘operation omitted’, with 44 tasks assigned this error (92%). Forty-four percent (21/47) and 32% (15/47) were assigned medium level of probability and medium level of criticality by the researchers, respectively. Key approaches stated for remedying these medium risk and medium probability errors were linked to training and provision of a checklist for NGT feeding tasks (see Additional file 1*:* Table S2).

## Discussion

Understanding how humans interact with elements of a system, such as technologies, is important in designing fully functional, effective and safe systems [16, 20]. Patient safety, in the healthcare setting, largely depends on carefully thought out ergonomics of the workplace processes implemented during care provision [32]. HFE, in the healthcare setting, has mostly focussed on the design of medical devices and other aspects of Information Technology for health to increase patient safety and reduce prevalence of medical errors [19, 33]. The focus of HFE in the healthcare is now shifting towards improving human wellbeing through identifying ways to improve work processes and reduce workloads, especially for already resource constrained settings such as infant inpatient settings in LMICs, and more so in Kenya [20]. In this paper we focus on the nursing aspect of care provision in inpatient neonatal care settings in Kenya.

Outcomes of inpatient care for small and sick infants are highly dependent on nursing care, with better outcomes correlated with low patient to nurse ratios [34]. Meeting recommended nurse to patient ratios is still a challenge in most low- and middle-income countries, including Kenya, leading to some tasks being informally shared with unskilled personnel and the infant’s mother. The nature of care required to improve patient outcomes in neonatal settings is intricate and time consuming. In this study, we explore the complexity of performing NGT feeding, one of the many key tasks that nurses do while providing care to small and sick infants in inpatient settings [35]. Reported sharing, supervision and risk levels of the 47 tasks in NGT feeding varied widely in this study, despite SMEs coming from fairly similar care settings serving the poor and this could be suggestive of differences in perception and practice.

If not undertaken correctly, NGT feeding can have many serious consequences [36]. However, the greatest risks lie during NGT insertion, a task that precedes NGT feeding. Perforations and incorrect placement of the NGT can occur [37]. The task of NGT insertion was recognised solely as a professional role by the SMEs, undertaken only by qualified and competent personnel and was not the focus of this study. Findings from this study indicated that ‘moderate risk’ was the highest level of risk assigned to tasks during NGT feeding. None of the tasks was deemed of ‘high risk’ by either the researchers or the SME, suggesting considerable consensus. The tasks identified as ‘medium risk’ can be targeted for specific training and/or supervision efforts to reduce risk and increase safety during NGT feeding.

We noted that sharing was mostly reported to be with the mothers. There is growing appreciation of the importance of involving family members and patients in care management, so called patient-family-centred care, as it positively influences neonatal care [38]. In high-income countries, this concept of care has developed over the years, placing parents/family members at the centre of care provision and promoting individualised and tailored health care services. Previous studies have shown that 80–95% of families prefer this kind of care, especially when teaching and discussion on the care of the infant occur at the bedside. [39] This highlights the health benefits of involving mothers or other family members in care of children, including neonates and has led to the development of recommendations on integrating patient-family-centred care by the American Academy of Paediatricians [40–42]. We also noted considerable heterogeneity in how sharing was reported by the experts. There were significant differences in the proportion of tasks reported as shared with either the mother, casual or students, despite the experts coming from fairly similar settings. Similar observations were also noted for reported supervision and risk levels of tasks. These differences could be due to the subjective nature of the perceptions and practice of task sharing by each expert in their respective settings and shows a gap in terms of clear and practical guidelines on how sharing of tasks should be implemented and which specific tasks should be shared, especially in the neonatal care context. One expert, for example, reported not sharing any of the tasks with the students, despite students being present in this setting, while the other expert (from the same setting) shared a considerable amount of tasks with the students (31.9%). We observed that some nurses were uncomfortable with or strongly opinionated against task sharing with students or other unqualified staff. These nurses often held policy or teaching roles that were somewhat removed from the real and practical frontline challenges in delivering nursing care in the context of limited human resources for health, among other challenges [43]. Anecdotal evidence, from other studies we are conducting in similar settings, also suggests that nurses tend to maintain a distinctive identity and therefore wield authority as to whom tasks can be shared with in their settings. This could explain why some experts will share tasks with the mother and not the students or the casuals, hence the heterogeneity in reported sharing proportions with the students, mothers and casuals. This shows a need for practical guidelines for task sharing currently not addressed in Kenya’s task sharing policy [12].

The use of HTA and SHERPA revealed the value of the HFE approach in eliciting these differences in perceptions that have direct effects on the quality and safety of NGT feeding. The involvement of mothers and unskilled personnel such as casual workers, in the provision of care for sick infants through task-sharing may help in ensuring that most, if not all the care that the neonate requires is provided. Tasks considered to be low risk can be reassigned to lower cadre workers within the neonatal setting, while high/medium risk tasks can be performed by the nurses; potentially managing the high workload that nurses have, especially in resource constrained settings like Kenya. In addition, nurses may have more time to provide the much needed critical care often associated with high/medium risk tasks. Those tasks reassigned should also be supervised in such a way as to reduce, if not eliminate, risks for undesirable outcomes during their implementation by the lower cadre. Careful consideration is necessary to ensure that the additional supervision responsibility on the nurses’ part does not become counterproductive. A delicate balance should be upheld to ensure that safety and quality of care is not compromised. Task sharing has the potential to help mitigate the health worker force shortages in LMICs, however, if undertaken without proper measures to ensure safety and quality, patient outcomes might be undesirable due to the potential likelihood of provision of low quality of care by whom the task is shared with. Therefore, provisions for standardised and detailed guidelines on training and supervision must be made for safe task-sharing and family-patient-centred care. Ergonomics methods have demonstrated to be useful in unpacking and understanding tasks in a way that can be applied to training and supervision needs, while at the same time highlighting focus areas of potential risks [18, 19]. During the course of the research, a novel way of annotating the HTA to show the task sharing and supervision was developed. This shows the flexibility of the method in being easily adaptable for new analyses.

A very small proportion (8.5%) of the 47 NGT feeding tasks were reported as often missed by the majority of the SMEs in this study. Contrary to the commonly used missed care definition however, and while fully aware of the risk of incorrect implementation of NGT feeding tasks, the SMEs did not consider a task as missed if it was performed by unskilled persons (casuals or mothers). Therefore, despite NGT feeding being an important aspect of care for sick neonates, nurses may often, knowingly, miss parts of the process or delegate to unskilled personnel. This can have significant effects on the recovery time and outcome of the infant [29, 44, 45]. Missed nursing care for sick infants has also been reported in other settings and is often related to support and comfort care [44]. Similarly, in this study we found that screening the bed/cot for privacy, talking to the infant and thanking the infant/mother were some of the tasks related to psychosocial elements of care that were reported as often missed by majority of the SME members.

This study has both strengths and limitations. We used two small groups of SMEs to unpack the complex nature of NGT feeding. Engaging SMEs in discussions on the selected aspects of NGT feeding implementation showed that there was an established implicit understanding of the task. These experts were chosen based on their experience rather than aiming to have a representative sample of care providers in public sector hospitals. The use of SMEs and involvement of small groups of experts in ergonomics methods research is the norm and is valued due to its efficiency in enabling in-depth focus on specific performance issues [21, 22]. The sample size may not be sufficient and lacks power statistically with regard to the task sharing aspect of the study. Our aim for doing this, however, was to gain a preliminary understanding of the norms and practices of task sharing in the SMEs’ care settings, and as part of ongoing work to understand the tasks done by nurses to inform future work on task sharing and measuring the work done by nurses [46]. These findings should therefore be interpreted with caution. We plan, in the near future, to share findings from a larger study exploring task sharing in neonatal settings in different hospitals in Kenya using a larger sample size. SME discussions were conducted in groups, which may have led to biased responses of individual experts when reporting on norms within their facilities and convergence of opinions. Furthermore, the provision of two experts from each of the four facilities in SME2, suggests they should not be thought of as independent respondents. Nonetheless, we report high heterogeneity in responses from individual experts in sharing, supervision and risk levels for NGT feeding. Further exploring the origins of the observed heterogeneity would have provided a better explanation to the observations, we, however, did not do this. Almost twice as many tasks were reported as highly supervised as those deemed as of medium risks, whether this increased demand for supervision had implications on the nurses’ workload was not further explored. Some tasks, such as ‘Insert syringe to tip of NG tube’ and ‘Pour feed into the syringe’ under the sub-goal ‘Perform NGT feeding’ were reported as often missed by minority of the SMEs yet the subsequent tasks were reported as never missed by all SMEs. This introduces some ambiguity given that the tasks are performed in sequence. One cannot, for example, ‘Allow the feed to flow by gravity’ if they missed pouring the feed into the syringe in the first place. Some of the noted discrepancies can best be disambiguated through observations. Observational work is often used to complement HTA in ergonomics methods, we plan, in future detailed reports, to share findings from in-depth ethnographic and other methods to explore missed care in Kenya.

To our knowledge, this is the first application of HFE methods to neonatal care research and healthcare in a low-resource setting. A significant number of systems used to report patient safety dwell on analysis of adverse events after they occur, however, there is a shift to focus more on proactive and progressive systems that enable identification of system weaknesses before tragic outcomes and thus avoiding failure modes [47]. Among such methods include HFMEA and HTA/SHERPA. In this study however, we chose to use HTA and SHERPA given our expertise and experience with the methods and their flexibility in their implementation across different teams. HFE methods have previously been shown to be valuable in highlighting patient safety issues during care provision [15, 19, 20]. In our setting, local researchers and SMEs found the methods engaging and easy to grasp [27, 48]. The SMEs welcomed the use of HFE to better understand and articulate the complexity of tasks that hitherto had been a form of implicit knowledge in Kenya making it difficult to share tasks or have standards that comprehensively guide task sharing. This positive experience is contrary to previous reports that healthcare professionals usually have an initial scepticism for these methods [49].

## Conclusion

Sharing tasks with lower cadre workers or even with a patient’s family in low-resourced healthcare settings may help ease the pressure of high workloads and nursing shortages. However, little is known about how task-sharing might impact safety and quality of care, particularly for neonatal patients where informal task-sharing seems common and not standardised. The novel use of HTA and SHERPA in this study to analyse NGT feeding in a low income neonatal setting revealed the value of these methods to describe the complexity and elicit quality and safety concerns in preforming routine nursing tasks. Our findings could lead to targeted, evidence-based local policy on reorganisation of tasks and detailed training and standard guidance for NGT feeding in neonatal care in LMIC as part of efforts to improve quality of care and reduce neonatal mortality. More widely HTA could help to formalise approaches to task sharing as well as identifying the training needs for non-professional carers, whereas SHERPA could help to assess the risks associated with sharing tasks. Together, these methods offer a way to help improve mortality rates in low-resource intensive settings.

## Additional files

Additional file 1: **Table S2.** Systematic human error reduction and prediction approach analysis table of the 47-nasogastric tube feeding tasks. (PDF 304 kb)

Additional file 2: HFE questionnaire. (DOCX 245 kb)

Additional file 3: **Figure S1.** Color coded NGT feeding HTA for missed task steps showing distribution of consensus among the SMEs for the tasks reported as often missed. (PNG 274 kb)

Additional file 4: **Table S1.** Systematic human error reduction and prediction approach (SHERPA) table of selected tasks assigned medium probability and criticality levels showing risks and supervision levels as reported by subject matter experts. (PDF 177 kb)
