# Supplementary material for: Neonatal nasogastric tube feeding in a low-resource African setting – using ergonomics methods to explore quality and safety issues in task sharing
Source: BMC Nurs. 2018 Nov 16;17:46. doi: 10.1186/s12912-018-0314-y (PMC6240229; doi:10.1186/s12912-018-0314-y)
Supplement: Supplementary file 1 — Table S2. Systematic human error reduction and prediction approach analysis table of the 47-nasogastric tube feeding tasks. (PDF 304 kb) [file 12912_2018_314_MOESM1_ESM.pdf]

Table S2: *Systematic human error reduction and prediction approach analysis table of the 47-nasogastric tube feeding tasks.*

SHERPA - NASOGASTRIC TUBE FEEDING								
Task step	Task	Error Mode	Error Description	Consequences	Recovery	Probability (P) 1 -Low, 2-Medium, 3-High	Criticality (C) 1-Low, 2-Medium, 3-High	Remedial Measures
1.1.1	Explain procedure and goal of procedure to mother	A9: Operation omitted	Procedure not explained to mother	Mother unaware of procedure and goal of procedure	NA	1	1	Continuous Medical Education (CME) on NGT feeding
1.1.2	Obtain parental consent/permission	A9: Operation omitted	Failure to obtain consent	Operation performed without consent	1.1.1	2	1	Continuous Medical Education (CME) on NGT feeding
1.2.1	Locate the trolley	A1: Operation too long	Taking too much time to locate the trolley	Trolley not located in time	NA	2	1	Have trolleys stored in a specific are
1.2.1	Locate the trolley	A7: Right operation on wrong object	Locate a trolley but a wrong one	Wrong trolley obtained	NA	2	2	Label trolleys; use a labelled trolley; use two trays or sterile field
1.2.1	Locate the trolley	A9: Operation omitted	Failure to locate trolley	No trolley for setting up feeding equipment	NA	2	1	Clear guidelines on use of trolleys for NGT feeding
1.2.2	Wipe entire trolley clean with spirit swab	A10: Operation incomplete	Failure to wipe the entire trolley	Trolley not entirely cleaned	1.2.1	2	2	Periodic reference to the procedure manual on how to clean trolleys
1.2.2	Wipe entire trolley clean with spirit swab	A9: Operation omitted	Failure to wipe the trolley	Trolley not cleaned	1.2.2	2	2	Periodic reference to the procedure manual on how to clean trolleys
1.2.3.1	Put milk jar, feed, calibrated cups and 2 syringes on the top shelf	A9: Operation omitted	Failure to put equipment on trolley	No feeding equipment on trolley	1.2.2	2	2	Provide a list of equipment needed for NGT feeding in preparation room
1.2.3.1	Put milk jar, feed, calibrated cups and 2 syringes on the top shelf	A10: Operation incomplete	Failure to set all the equipment required on the top shelf of the trolley	Some feeding equipment missing on trolley	1.2.2	2	2	Provide a list of equipment needed for NGT feeding in preparation room
1.2.3.2	Put cotton swabs, clean linen, receiver for gastric content and used items on the bottom shelf	A9: Operation omitted	Failure to put the equipment required on the bottom shelf of the trolley	Feeding equipment missing on trolley	1.2.3.1	2	2	Provide a list of equipment needed for NGT feeding in preparation room
1.2.3.2	Put cotton swabs, clean linen, receiver for gastric	A10: Operation incomplete	Failure to set all the equipment required on	Some feeding equipment missing on trolley	1.2.3.1	2	2	Provide a list of equipment needed for NGT feeding in preparation room

	content and used items on the bottom shelf		the bottom shelf of the trolley					
1.2.4	Confirm if all equipment/materials are on trolley	C1: Check omitted	Failure to check if the equipment are on the trolley	Equipment not checked if on the trolley therefore no feeding equipment on the trolley	1.2.3.1, 1.2.3.2	2	2	Provide a list of equipment needed for NGT feeding in preparation room
1.2.4	Confirm if all equipment/materials are on trolley	C2: Check incomplete	Failure to check if all the equipment are on the trolley	Not all equipment checked if on the trolley therefore some might be missing	1.2.3.1, 1.2.3.3	2	2	Provide a list of equipment needed for NGT feeding in preparation room
1.3.1	Switch on lights, raise window curtains	A9: Operation omitted	Failure to switch on lights, raise window curtains	Not enough light in the room	NA	1	1	Assign this activity to subordinate in charge of cleaning
1.3.2	Screen bed/cot for privacy	A9: Operation omitted	Failure to screen bed/cot for privacy	No privacy attained	NA	1	1	Assign this activity to subordinate in charge of cleaning
1.3.3	Remove any obstacles around baby's cot	A9: Operation omitted	Failure to remove any obstacles around baby's cot	Unsafe feeding environment due to clutter or obstacles	NA	1	1	Assign this activity to subordinate in charge of cleaning
1.4.1	Position baby to a slightly elevated position (supine)	A9: Operation omitted	Failure to position baby correctly before feeding	Baby not positioned properly for feeding	NA	1	1	CMEs on NGT feeding
1.4.2	Clean/wipe any oral nasal secretions	A9: Operation omitted	Oral/nasal secretions not cleaned	Possible contamination of feed	NA	2	2	CMEs on NGT feeding
1.4.3.1	Touch the baby	A9: Operation omitted	Failure to touch baby		NA	1	1	CMEs on NGT feeding and importance of comfort care to patients
1.4.3.2	Talk to the baby	A9: Operation omitted	Failure to talk to baby		NA	1	1	CMEs on NGT feeding and the importance of communication (verbal/nor-verbal) while providing care
1.4.4.1	Kink NG-tube	A9: Operation omitted	Failure to kink NGT	Tube not kinked, therefore air enters into the tube	NA	2	1	CMEs on NGT feeding
1.4.4.2	Open tip of the tube	A9: Operation omitted	Failure to open tip of tube	Tip of tube not open therefore cannot insert syringe	NA	1	1	CMEs on NGT feeding
1.4.4.3	Attach syringe	A9: Operation omitted	Failure to attach syringe	Syringe not attached	NA	1	1	CMEs on NGT feeding
1.4.4.4	Remove kink	A9: Operation omitted	Failure to remove kink	Kink not removed therefore cannot pull plunger to draw gastric contents for assessment	NA	1	1	CMEs on NGT feeding

1.4.4.5	Pull plunger	A9: Operation omitted	Failure to pull plunger	Plunger not pulled therefore cannot withdraw stomach contents		2	2	CMEs on NGT feeding
1.4.4.6	Assess gastric content type and volume	A9: Operation omitted	Gastric assessment not done	Incorrect measure of feed given/Feed given when not necessary	NA	2	2	CMEs on NGT feeding
1.4.4.7	Slowly push back gastric contents	A9: Operation omitted	Failure to push gastric contents back					CMEs on NGT feeding
1.4.4.8	Kink NG-tube	A9: Operation omitted	Failure to kink NGT	Tube not kinked, therefore air enters into the tube	NA	2	1	
1.4.4.9	Remove syringe	A9: Operation omitted	Failure to remove syringe	Syringe not removed therefore cannot replace lid	1.4.4.8	1	1	CMEs on NGT feeding
1.4.4.10	Replace NG-tube lid	A9: Operation omitted	Failure to replace NGT lid	Tube not closed, therefore air enters the tube	1.4.4.9	1	1	CMEs on NGT feeding
2.1	Review/check prescribed feed to determine amount of feed	C1: Check omitted	Failure to review/check prescribed feed to determine amount of feed	Feed prescribed and amount not checked	2.6	2	2	Avail a list of steps to be followed while conducting NGT feeding
2.2	Remove plunger from syringe	A9: Operation omitted	Failure to remove plunger from syringe	Plunger retained in syringe, cannot pour feed into the barrel	2.6	1	1	Avail a list of steps to be followed while conducting NGT feeding
2.3	Kink tube	A9: Operation omitted	Failure to kink NGT	Tube not kinked, therefore air enters into the tube	2.2	2	1	Avail a list of steps to be followed while conducting NGT feeding
2.4	Remove tip covering NGT	A9: Operation omitted	Failure to remove tip covering NGT	Tube not opened, unable to insert syringe	2.3	2	1	Avail a list of steps to be followed while conducting NGT feeding
2.5	Insert syringe to tip of NGT	A9: Operation omitted	Failure to insert syringe	Syringe not inserted to the tip of NGT therefore cannot pour feed into the barrel of the syringe	2.7	1	1	Avail a list of steps to be followed while conducting NGT feeding
2.6	Pour feed into syringe barrel	A9: Operation omitted	Failure to pour feed	No feed poured therefore cannot assess speed of flow	2.1	1	1	Avail a list of steps to be followed while conducting NGT feeding
2.7	Release kink	A9: Operation omitted	Failure to release kink	Tube kinked, feed unable to flow	2.8	1	1	Avail a list of steps to be followed while conducting NGT feeding
2.8	Allow feed to flow by gravity	A9: Operation omitted	Failure to allow feed to flow by gravity	Feed not flowing	2.9	2	2	Avail a list of steps to be followed while conducting NGT feeding
2.9	Check if feed is flowing	C1: Check omitted	Failure to check if feed is flowing	Feed not flowing	2.8	2	2	Avail a list of steps to be followed while conducting NGT feeding
2.10	Detach syringe from NGT	A9: Operation omitted	Failure to detach syringe from NGT	Unable to close tip of tube	2.12	1	1	Avail a list of steps to be followed while conducting NGT feeding
2.11	Close tip of NGT	A9: Operation omitted	Failure to close tip of tube	Tube left open therefore air enters the tube	3.2	1	1	Avail a list of steps to be followed while conducting NGT feeding

3.1	Wipe any secretions or spillages	A9: Operation omitted	Failure to wipe secretions and spillages	Baby left untidy	NA	1	1	Avail a list of steps to be followed while conducting NGT feeding
3.2	Secure NGT using adhesive tape	A9: Operation omitted	Failure to secure NGT	Risk of dislodging NGT	3.3	2	2	Avail a list of steps to be followed while conducting NGT feeding
3.3	Reposition baby to lateral position	A9: Operation omitted	Failure to reposition baby	Baby wrongly positioned, risk of regurgitation complications	3.4	2	2	Avail a list of steps to be followed while conducting NGT feeding
3.4	Cover baby	A9: Operation omitted	Failure to cover baby	Baby exposed to cold	NA	1	1	Avail a list of steps to be followed while conducting NGT feeding
3.5	Thank baby and mother	A9: Operation omitted	Failure to thank baby and mother		NA	1	1	Avail a list of steps to be followed while conducting NGT feeding
4.1	Take all dirty utensils to disinfection corner/room	A9: Operation omitted	Failure to take all dirty utensils to disinfection corner/room	Dirty utensils not taken to disinfection corner/room	NA	1	1	Avail a list of steps to be followed while conducting NGT feeding
4.2	Discard wastes according to waste disposal guidelines	A9: Operation omitted	Failure to discard waste according to waste disposal guidelines	Waste not discarded as per policy, risk of contamination and infection	NA	2	2	Avail a list of steps to be followed while conducting NGT feeding
4.3	Clean trolley	A9: Operation omitted	Failure to clean trolley	Trolley not cleaned, risk of cross contamination	NA	2	2	Avail a list of steps to be followed while conducting NGT feeding
4.4	Return trolley to procedure room	A9: Operation omitted	Failure to return trolley to procedure room		NA	2	2	Avail a list of steps to be followed while conducting NGT feeding
5.1	Document procedure in feeding chart	A9: Operation omitted	Documentation not done in feeding chart	No records of the procedure in the feeding chart for reference	5.2	2	2	Avail a list of steps to be followed while conducting NGT feeding
5.2	Document procedure in cardex	A9: Operation omitted	Documentation not done in cardex	No records of the procedure in the cardex for reference	NA	2	2	Avail a list of steps to be followed while conducting NGT feeding
