# Supplementary material for: Neonatal nasogastric tube feeding in a low-resource African setting – using ergonomics methods to explore quality and safety issues in task sharing
Source: BMC Nurs. 2018 Nov 16;17:46. doi: 10.1186/s12912-018-0314-y (PMC6240229; doi:10.1186/s12912-018-0314-y)
Supplement: Supplementary file 2 — HFE questionnaire. (DOCX 245 kb) [file 12912_2018_314_MOESM2_ESM.docx]

**KEMRI Wellcome Trust Research Programme: Information Sheet**

**Neonatal Nursing Task Analysis: Describing the tasks done by nurses caring for sick newborns**

**in Kenyan health-facilities**

| Institution | Investigators |
| --- | --- |
| KEMRI Wellcome Trust | Dr Georgina Murphy, Dr David Gathara, Mr Gregory Omondi, Dr Nancy  Abuya, Mr Steve Adala, Prof Mike English |
| University of Oxford | Dr Georgina Murphy |
| University of Southampton | Prof Neville Stanton |
| National Nurses Association of Kenya | Ms Leah Mbuya |

**Introduction**

Although Kenya is making progress in reducing deaths among children, there has been less

reduction in deaths among newborn babies. Many of these deaths could be prevented with high

quality inpatient care delivered by skilled nurses. However, there are often shortages in the number

of nurses needed. These shortages put nurses under strain to deliver adequate care and may lead to

nurses consciously or unconsciously prioritising certain tasks and care they provide to patients,

leading to varied quality with which the tasks are performed and some tasks being left undone (missed

care).

**Who is carrying out this study and what is this study about?**

In this study, KEMRI Wellcome Trust Research Program (KWTRP), the Ministry of

Health (Department of Nursing) and the Kenya Paediatric Association (KPA) aim to gain insight

into the valuable work done by nurses when providing care to sick newborns and will involve

600 nurses across the country. We want to characterise neonatal nursing tasks by understanding how

nurses report spending their working time, which tasks are considered critical and/or difficult, with

whom tasks are shared and how often tasks are done (or left undone). The study will give voice to

nurses to describe the work they do and provide valuable information to policymakers and the nursing

community.

**Why do you want to talk to me and what does it involve?**

Nurses who are CURRENTLY or have provided care in the last 2 years to inpatient sick

newborns are being invited to partake in this survey across Kenya using questionnaires that are being

distributed and collected by a local nurse coordinator. We feel that your experience as person

providing inpatient care to sick newborns can contribute much to our understanding and

knowledge on how care is delivered, the difficulty and importance of various tasks, who else apart

from nurses performs some tasks, and how often tasks may have to be left undone within the

newborn unit. The information collected through this questionnaire will be anonymous and we

will ensure that your identity is protected. Please do not write your name or any other personal

information on the questionnaire. Please fill this questionnaire and return it to the person who

gave it to you as soon as you have completed it. It is fine to take it away and return it once

complete or within one week. We will also request your phone number so that we can use it to contact about one out of five participants, randomly chosen, later to confirm their understanding of the questionnaire.

All participation in research is voluntary; you can decide freely whether or not you want to participate.

If you do agree first, you can change your mind at any time without giving a reason without

any consequences.

**Are there any risks or disadvantages to me taking part?**

Any potential risk of describing the tasks done by you when caring for sick newborns will be

mitigated (taken care of) by ensuring confidentiality of results and not recording your name on the

questionnaire or resulting data. We will ensure that your information is kept confidentially and

securely. Completing this questionnaire will take you approximately 40 minutes.

**Are there any advantages to me for taking part?**

We will not provide you any compensation for your time but issue you with a printed copy of

the Kenya Basic Paediatric Protocols booklet that we hope you will find useful for reference in your

daily work. By participating in this study, you will help provide a better understanding of how nurses

spend their time, the tasks provided in the newborn unit, their perceived importance and which of

these tasks may be left undone. These findings may help other people in Kenya and elsewhere in

the future, for example through developing new health policies on staffing norms for nursing or for

helping identify how best to help nurses to provide neonatal care.

**Who will have access to the information I give?**

All of our documents/ recordings are stored securely in locked cabinets and on password protected

computers. We are collecting information from nurses across Kenya and will make a report based on

all these questionnaires. We will not record your name and only summary results from across large

groups of nurses will be presented. Therefore, your identity will not be inferred from the results. The

knowledge gained from this research will be shared in summary form with Ministry of Health, Health

Professional Regulatory Boards and Councils, Health Professional Associations and the wider

international community through publications and presentations.

In future, information collected or generated during this study may be used to support new research by

other researchers in Kenya on staffing for nursing or for helping identify how best to help nurses to

provide neonatal care. In all cases, we will only share information with other researchers in ways that

do not reveal individual participants’ identities. In the questionnaire we are asking you to fill there will

be no information that could identify you on the questionnaire at all. If anyone does wish to reuse the

information from the questionnaires this must first be approved by a local or national expert

committee to make sure that the interests of participants and their communities are protected.

**Who has allowed this research to take place?**

All research at KEMRI has to be approved before it begins by several national committees who

look carefully at planned work. They must agree that the research is important, relevant to

Kenya and follows nationally and internationally agreed research guidelines. This includes ensuring that

all participants’ safety and rights are respected.

**What will happen if I refuse to participate?**

All participation in research is voluntary. You are free to decide if you want to take part or not. If

you do agree you can change your mind at any time without any consequences. As we are asking

you to take a questionnaire away with you and return it we will assume you are agreeing to give

information to the study when you return the questionnaire. If you do not return the questionnaire we

will assume you do not want to give information to the study.

**What if I have any questions?**

If you have any further questions about the questionnaire or study, please do not hesitate to ask your

local coordinator/survey staff or to contact the research team using the contacts below:

Main contact person: Gregory B. Omondi, KEMRI Wellcome Trust Research Programme,

P.O. Box 43640 – 00100 Nairobi, Kenya; Telephone: [0711823487]
Email: GOmondi@kemriwellcome.org.

Dr David Gathara, KEMRI Wellcome Trust Research Programme, P.O. Box 43640 – 00100

Nairobi, Kenya; Telephone: [0730162000] or 20 2719936 Email: Dgathara@kemri-wellcome.org

For specific information about the involvement of National Nurses Association of Kenya (NNAK)

please contact Leah Mbuya, NNAK P.O. BOX 49422-00100, Nairobi, Kenya; Telephone:

[0720740882] Email: leah@nnak.or.ke

If you want to ask someone independent anything about this research, please contact:

Community Liaison Manager, KEMRI Wellcome Trust Research Programme, P.O. Box 230,

Kilifi. Telephone: 041 7522 063, Mobile 0723 342 780 or 0705 154 386

And

The Head – KEMRI Scientific and Ethics Review Unit, P. O. BOX 54840-00200, Nairobi, Tel

number: 0717 719477 Email address: [seru@kemri.org](mailto:seru@kemri.org)

**Neonatal Nursing Task Analysis (NNTA) Study
Human Factors Ergonomics Workshop
Allocation Function Exercise**

**Introduction**

The presence of major courses of neonatal deaths and long-term morbidity such as pre-term birth, intra-uterine growth restriction, birth asphyxia and infection may necessitate the need for use of a nasogastric tube to facilitate feeding and medication administration. We are interested in finding out which task steps nurses share and with whom they share them in the process of feeding a baby via a nasogastric tube and the supervision aspects as well as supervision levels of such shared task steps.

**Instructions**

1. All the materials you will need for this exercise have been provided (highlighters, writing pads/notebooks, pens, stickers, HTA (*Hierarchical Task Analysis)* charts)
2. Please indicate in the boxes provided on a separate document containing the keys, where you are currently providing care to sick and hospitalized newborns (**newborn unit (NBU), maternity or paediatric unit/ward)**.
3. For each of the task steps in the NGT feeding HTA (*Hierarchical Task Analysis*), please indicate by **shading in the task box on the separate chart provided,** with who you share the task step **using the key of the color codes provided** (Blue-shared with the student; Green – shared with the mother; Yellow – shared with the casual worker; Orange – shared with the patient attendant). If you do not share the task step kindly shade it red as indicated in the key.


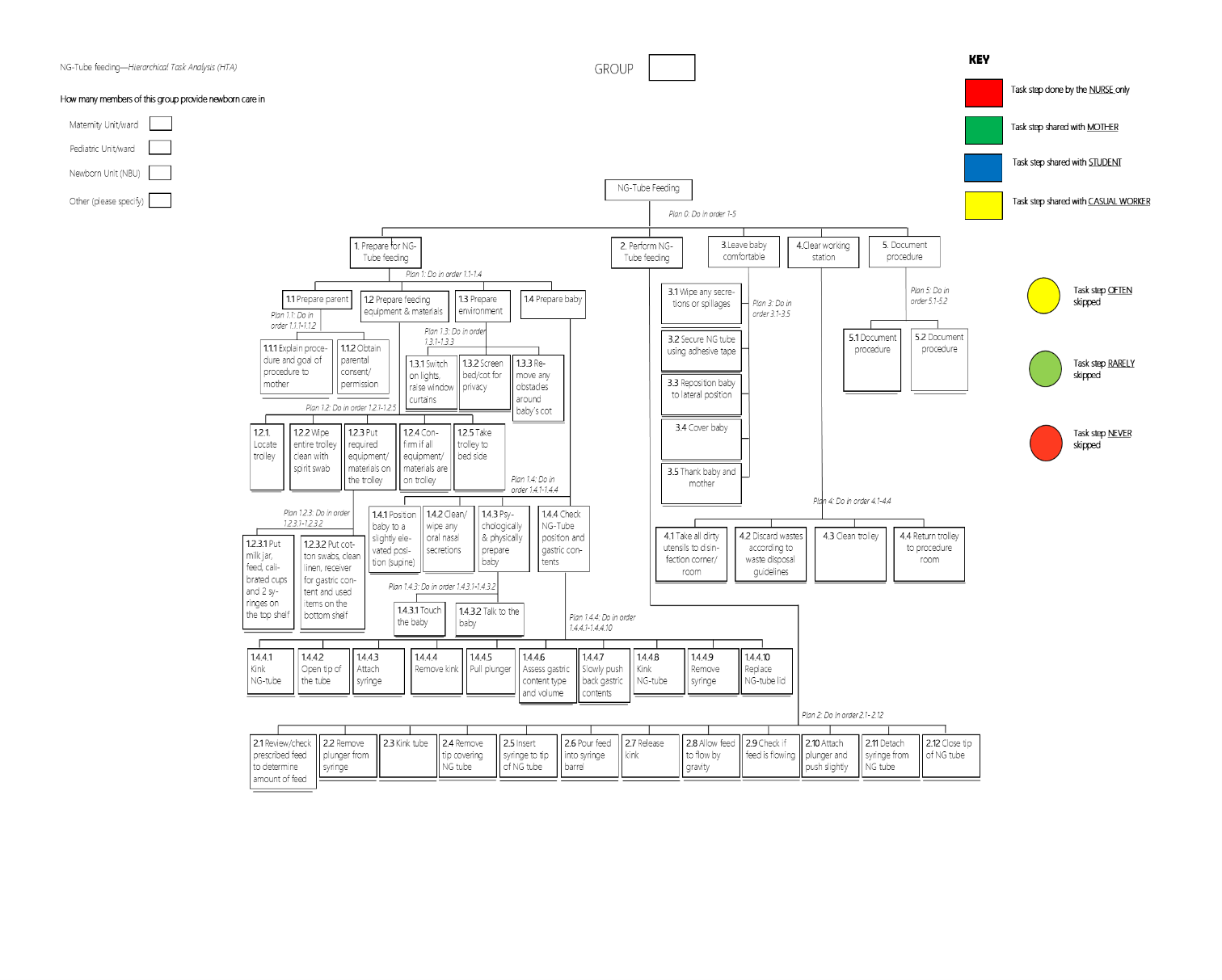


1. Kindly also indicate, by putting a sticker on each task box, which task steps are **often skipped/omitted, rarely skipped/missed and never skipped/missed** by **sticking the appropriate color of sticker on the box containing that particular tasks step**. *Use the key provided for frequency of task step omission as shown in the figure above and the stickers provided to mark the tasks appropriately.*
2. Please indicate which task steps you supervise and the level of supervision involved in such a step bearing in mind who you share the task step with. Do this by first identifying the task step that you supervise using the task step number i.e. in this exercise, the task step number for the task “Obtain parental consent/permission” is **1.1.2,** then indicate the level of supervision (high, moderate or low) of that task followed by the level of risk (high, medium, low).
   Record your results in the table below as per the example shown.

*Example:*

| **Task step** | **Supervision level** | **Risk level** |
| --- | --- | --- |
| *1.1.2* | *Low* | *Medium* |
|  |  |  |

| **Task step** | **Supervision level** | **Risk level** |
| --- | --- | --- |
|  |  |  |
|  |  |  |
|  |  |  |
|  |  |  |
|  |  |  |
|  |  |  |
|  |  |  |
|  |  |  |
|  |  |  |
|  |  |  |
|  |  |  |
|  |  |  |
|  |  |  |
|  |  |  |
|  |  |  |
|  |  |  |
|  |  |  |
|  |  |  |
|  |  |  |
|  |  |  |
|  |  |  |
|  |  |  |
|  |  |  |
|  |  |  |
|  |  |  |
|  |  |  |
|  |  |  |
|  |  |  |
|  |  |  |
|  |  |  |
|  |  |  |
|  |  |  |
|  |  |  |
|  |  |  |
|  |  |  |
|  |  |  |
|  |  |  |
|  |  |  |
|  |  |  |
|  |  |  |
|  |  |  |
|  |  |  |
|  |  |  |
|  |  |  |
|  |  |  |
|  |  |  |
|  |  |  |
|  |  |  |
|  |  |  |
|  |  |  |
|  |  |  |
|  |  |  |
|  |  |  |
|  |  |  |
|  |  |  |
|  |  |  |
|  |  |  |
|  |  |  |
|  |  |  |
|  |  |  |
|  |  |  |
|  |  |  |
|  |  |  |
|  |  |  |
