# Supplementary figures and images for: Neonatal nasogastric tube feeding in a low-resource African setting – using ergonomics methods to explore quality and safety issues in task sharing
Source: BMC Nurs. 2018 Nov 16;17:46. doi: 10.1186/s12912-018-0314-y (PMC6240229; doi:10.1186/s12912-018-0314-y)

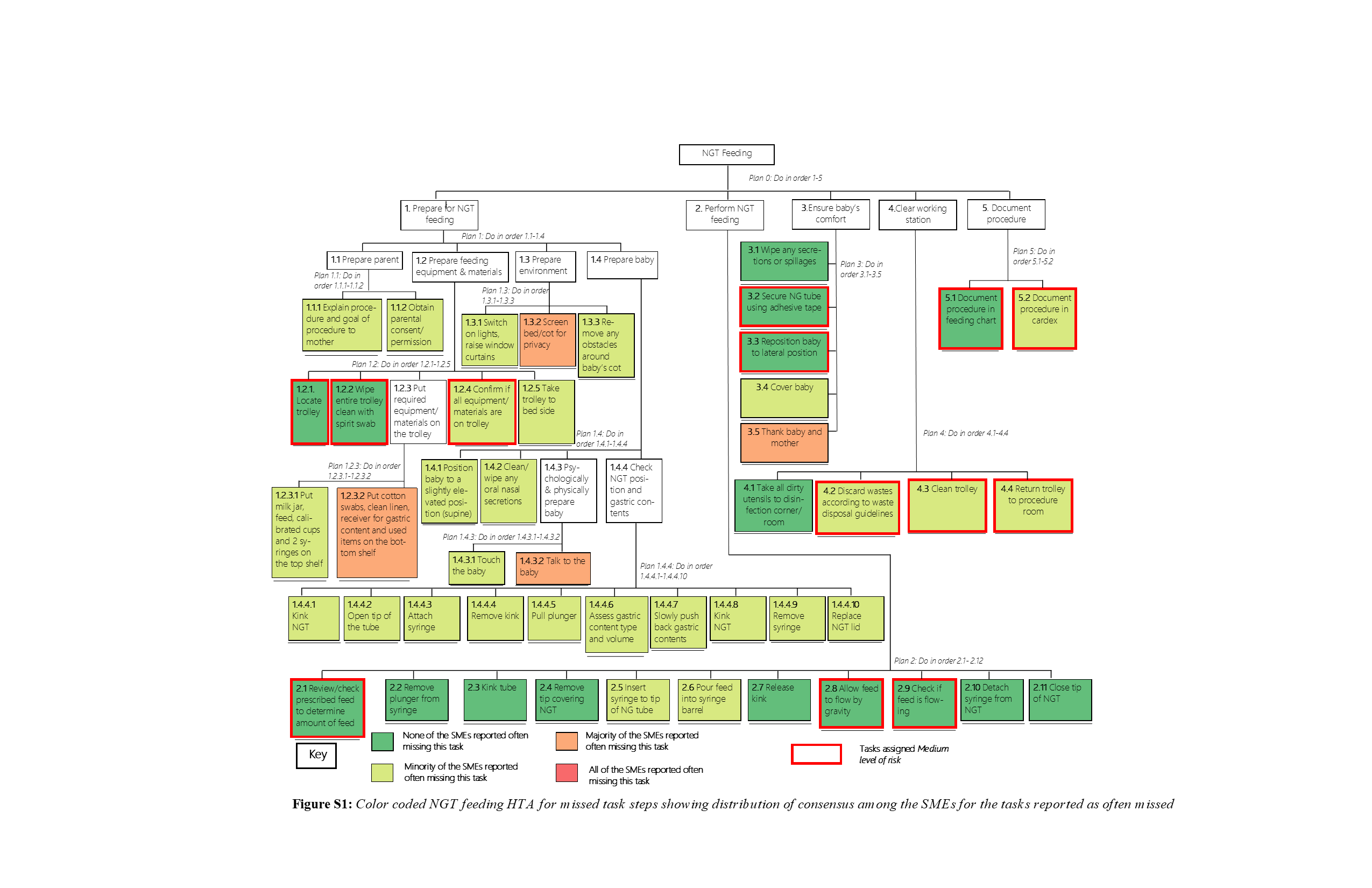

Supplement: Supplementary file 3 — Figure S1. Color coded NGT feeding HTA for missed task steps showing distribution of consensus among the SMEs for the tasks reported as often missed. (PNG 274 kb) [file 12912_2018_314_MOESM3_ESM.png]
