# Supplementary material for: Neonatal nasogastric tube feeding in a low-resource African setting – using ergonomics methods to explore quality and safety issues in task sharing
Source: BMC Nurs. 2018 Nov 16;17:46. doi: 10.1186/s12912-018-0314-y (PMC6240229; doi:10.1186/s12912-018-0314-y)
Supplement: Supplementary file 4 — Table S1. Systematic human error reduction and prediction approach (SHERPA) table of selected tasks assigned medium probability and criticality levels showing risks and supervision levels as reported by subject matter experts. (PDF 177 kb) [file 12912_2018_314_MOESM4_ESM.pdf]

Table S1: *Systematic human error reduction and prediction approach (SHERPA) table of selected tasks assigned medium probability and criticality levels showing risks and supervision levels as reported by subject matter experts.*

	Task	Shared with	Number of SMEs reporting sharing of this task with mother/casual	Number of SMEs reporting this task was often missed	Error mode & description	Consequences	Risk level as reported by SMEs	Supervision level	Remedial Measures
1.2.1	Locate the trolley	Casual	2	0	A7: Right operation on wrong object; Locate a trolley but a wrong one	Wrong trolley obtained	Medium	Medium	Label trolleys; use a labelled trolley; use two trays or sterile field
1.2.2	Wipe entire trolley clean with spirit swab	Mother	0	0	A9: Operation omitted; Failure to wipe the trolley	Trolley not cleaned	Medium	Medium	Periodic reference to the procedure manual on how to clean trolleys
1.2.3.1	Put milk jar, feed, calibrated cups and 2 syringes on the top shelf	Mother /casual	2/2	1	A9: Operation omitted; Failure to put equipment on trolley	No feeding equipment on trolley	Low	High	Provide a list of equipment needed for NGT feeding in preparation room
1.2.3.2	Put cotton swabs, clean linen, receiver for gastric content and used items on the bottom shelf	Mother	1	4	A9: Operation omitted; Failure to put the equipment required on the bottom shelf of the trolley	Feeding equipment missing on trolley	Low	Low	Provide a list of equipment needed for NGT feeding in preparation room
1.2.4	Confirm if all equipment/materials are on trolley	Casual	1	1	C1: Check omitted; Failure to check if the equipment is on the trolley	Equipment not checked if on the trolley therefore no feeding equipment on the trolley	Medium	Low	Provide a list of equipment needed for NGT feeding in preparation room
1.4.2	Clean/wipe any oral nasal secretions	Mother	5	2	A9: Operation omitted; Oral/nasal secretions not cleaned	Possible contamination of feed	Low	High	CMEs on NGT feeding
1.4.4.5	Pull plunger	Mother	3	1	A9: Operation omitted; Failure to pull plunger	Plunger not pulled therefore cannot withdraw stomach contents	Low	Medium	
1.4.4.6	Assess gastric content type and volume	Mother	4	2	A9: Operation omitted; Gastric assessment not done	Incorrect measure of feed given/Feed given when not necessary	Low	High	CMEs on NGT feeding
1.4.4.7	Slowly push back gastric contents	Mother	4	2	A9: Operation omitted; Failure to		Low	High	

					push gastric contents back				
2.1	Review/check prescribed feed to determine amount of feed	Mother	1	0	C1: Check omitted; Failure to review/check prescribed feed to determine amount of feed	Feed prescribed and amount not checked	Medium	High	Avail a list of steps to be followed while conducting NGT feeding
2.8	Allow feed to flow by gravity	Mother	6	0	A9: Operation omitted; Failure to allow feed to flow by gravity	Feed not flowing	Medium	High	Avail a list of steps to be followed while conducting NGT feeding
2.9	Check if feed is flowing	Mother	5	0	C1: Check omitted; Failure to check if feed is flowing	Feed not flowing	Medium	High	Avail a list of steps to be followed while conducting NGT feeding
3.2	Secure NGT using adhesive tape	Mother	3	0	A9: Operation omitted; Failure to secure NGT	Risk of dislodging NGT	Medium	Medium	Avail a list of steps to be followed while conducting NGT feeding
3.3	Reposition baby to lateral position	Mother	5	0	A9: Operation omitted; Failure to reposition baby	Baby wrongly positioned, risk of regurgitation complications	Medium	High	Avail a list of steps to be followed while conducting NGT feeding
4.2	Discard wastes according to waste disposal guidelines	Mother /casual	2/4	1	A9: Operation omitted; Failure to discard waste according to waste disposal guidelines	Waste not discarded as per policy, risk of contamination and infection	Medium	Medium	Avail a list of steps to be followed while conducting NGT feeding
4.3	Clean trolley	Casual	3	2	A9: Operation omitted; Failure to clean trolley	Trolley not cleaned, risk of cross contamination	Medium	Medium	Avail a list of steps to be followed while conducting NGT feeding
4.4	Return trolley to procedure room	Casual	3	3	A9: Operation omitted; Failure to return trolley to procedure room		Medium	Low	Avail a list of steps to be followed while conducting NGT feeding
5.1	Document procedure in feeding chart	Mother	4	0	A9: Operation omitted; Documentation not done in feeding chart	No records of the procedure in the feeding chart for reference	Medium	High	Avail a list of steps to be followed while conducting NGT feeding
5.2	Document procedure in cardex	Mother	1	1	A9: Operation omitted; Documentation not done in cardex	No records of the procedure in the cardex for reference	Medium	Medium	Avail a list of steps to be followed while conducting NGT feeding

KEY

Colour	Meaning
Green	Tasks reported as shared with mothers/casuals by half or more of the SME
Yellow	Tasks reported as being missed by half or more of the SMEs
